# Stent-Associated Coronary Artery Aneurysm Treated With Interposition Graft and Branch Reimplantation

**DOI:** 10.1016/j.jaccas.2026.109554

**Published:** 2026-08-12

**Authors:** Jose L. Diz Ferre, Mahmoud Alshneikat, Faisal G. Bakaeen, Gosta B. Pettersson, Rishabh Khurana, Anthony L. Zaki, Edward G. Soltesz, Eric E. Roselli, Paulino Alvarez, Shinya Unai

**Affiliations:** a Department of Thoracic and Cardiovascular Surgery, Cleveland Clinic, Cleveland, Ohio, USA; b Section of Cardiovascular Imaging, Cleveland Clinic, Cleveland, Ohio, USA; c Kaufman Center for Heart Failure, Cleveland Clinic, Cleveland, Ohio, USA

**Keywords:** acute heart failure, coronary artery bypass, stents

## Abstract

**Background:**

Coronary artery aneurysms have an incidence of 0.3% to 5.3% following percutaneous transluminal coronary angioplasty.

**Case Summary:**

A 75-year-old woman presented with dyspnea on exertion and chest discomfort. In 2004 she underwent percutaneous coronary intervention to the left anterior descending artery (LAD) and, in 2010, a ST-segment elevation myocardial infarction was treated with another LAD stent. On this admission, catheterization revealed a large proximal LAD coronary aneurysm at the site where the last stent was placed. She underwent successful LAD aneurysm repair with end-to-end saphenous vein graft interposition and end-to-side bypass to the diagonal branches.

**Discussion:**

The most common surgical procedures are ligation and coronary artery bypass graft. In our case, the diagonal branches were too small to perform epicardial bypasses.

**Take-Home Messages:**

Repair of coronary aneurysm with interposition of saphenous vein graft re-establishes flow to the main vessel and tributaries. Adequate exposure and meticulous techniques are essential for success.

## Visual Summary

**Figure:**
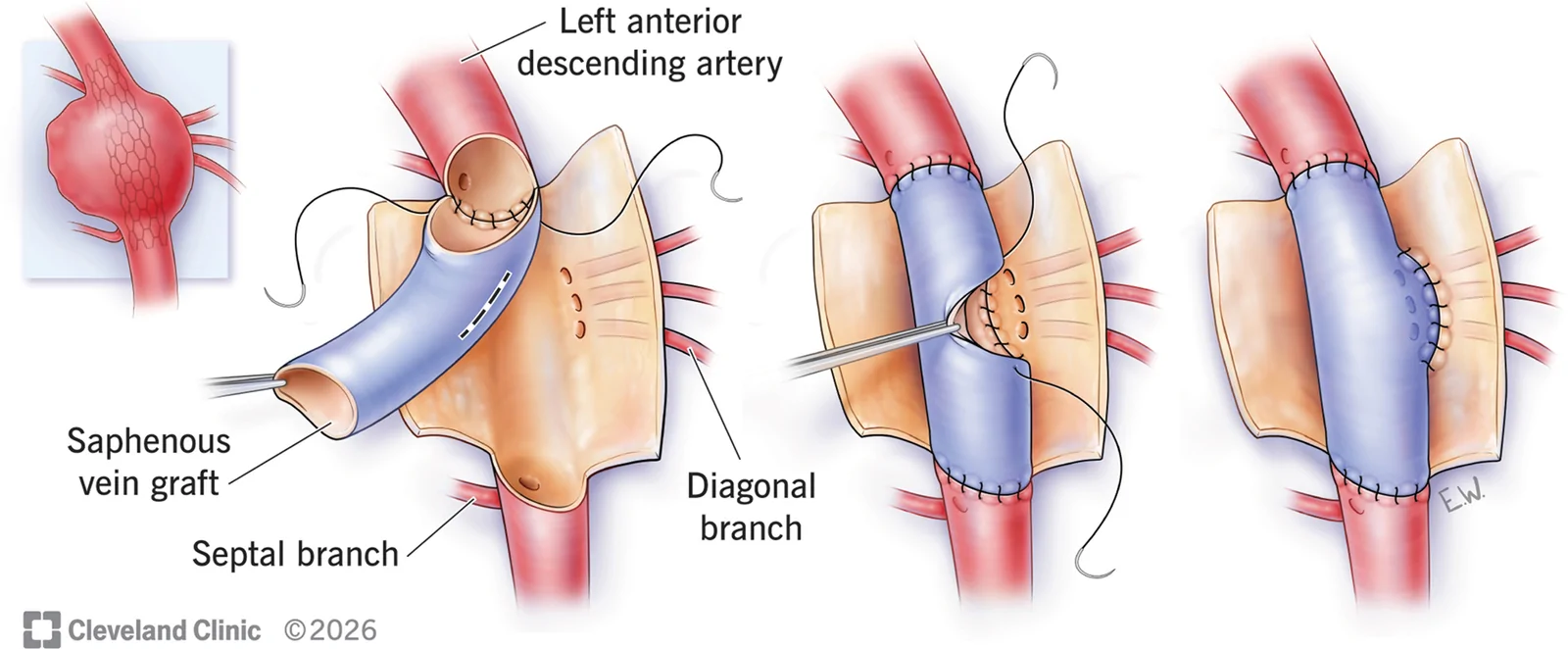

## History of Presentation

A 75-year-old woman presented with multiple episodes of severe central chest pain, described as so intense that she “thought I would die” and was unable to get up off the floor. Stress test revealed mild ischemia and a small scar and area of hibernating myocardium in the left anterior descending artery (LAD) territory.

## Past Medical History

Her past medical history included mild stage B heart failure due to ischemic cardiomyopathy and breast cancer treated with right partial mastectomy and accelerated partial breast radiation with a total dose of 2,850 cGy in 5 fractions. Over the past year, she also was treated for a second malignancy, a mucosa-associated lymphoid tissue non-Hodgkin lymphoma involving the trachea. Treatment included bronchoscopy, laser ablation, and radiation therapy of the tracheal mass, and 6 cycles of chemotherapy with bendamustine plus rituximab complicated by prolonged neutropenia. She also had a positive lupus anticoagulant.

More than 2 decades ago, she was diagnosed with coronary artery disease manifested in left arm pain. Percutaneous coronary intervention (PCI) was performed with a drug-eluting stent (DES) to proximal LAD. After an episode of epistaxis and interrupted antiplatelet therapy, she returned 6 years later with an ST-segment elevation myocardial infarction and 100% LAD occlusion at the site of the previous stent. This was treated with PCI, including transluminal extraction atherectomy.

## Differential Diagnosis

Differential diagnosis includes acute on chronic heart failure and stable angina.

## Investigations

She did not have signs of heart failure. The electrocardiogram showed normal sinus rhythm and left axis deviation along with left ventricular hypertrophy and altered repolarization. Reviewing an old, computed tomography scan from 2021, a mild dilatation of the LAD at the site of the proximal LAD stent was observed (Figure 1).

**Figure 1:**
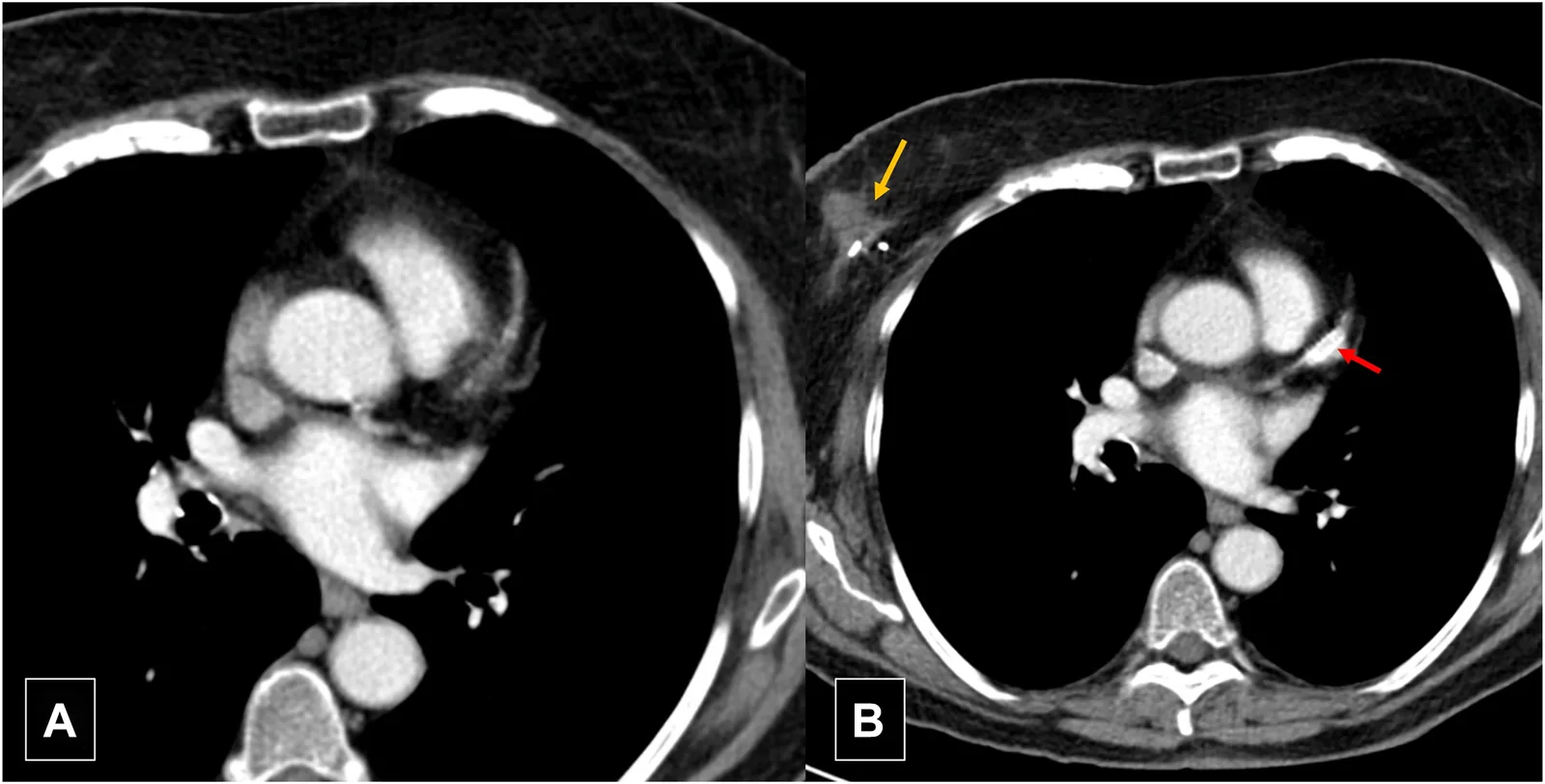
Preoperative Computed Tomography (A and B) A previous, 4-year-old nongated contrast-enhanced computed tomography scan of the chest depicts the left anterior descending coronary artery mildly dilated with stent in place (red arrow). In addition, postsurgical and radiation changes can be seen in the right breast (orange arrow in B).

Positron emission tomography verified mild (<10%) ischemia, evidence of a small (<10%) scar, and a medium (10% to 20%) area of hibernating myocardium in the LAD territory. Echocardiography showed the left ventricle with a normal size but with mildly decreased systolic function. Diagnostic catheterization revealed a large aneurysm in the proximal LAD at the site of the prior stent (Figure 2). Computed tomography angiography showed a proximal LAD stent with a mild in-stent stenosis proximally and a surrounding saccular coronary aneurysm (maximal diameter of 13 mm). There was a mild amount of predominantly calcified plaque in the proximal LAD and just distal to the stent and aneurysm was observed with a minimal luminal diameter stenosis (1% to 24%) (Figure 3).

**Figure 2:**
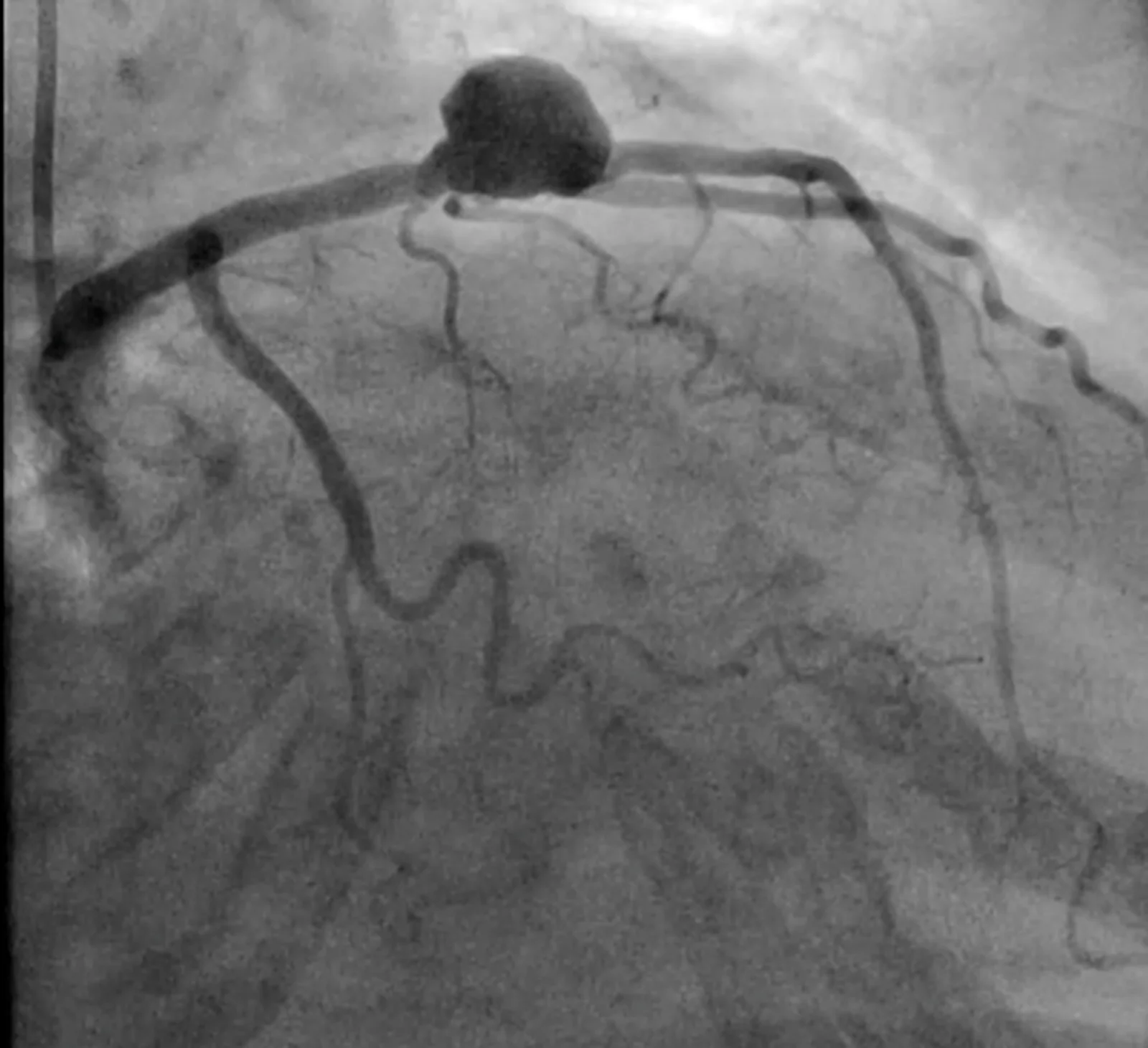
Diagnostic Coronary Angiography Demonstrating the Large Proximal Aneurysm in the Left Anterior Descending Artery

**Figure 3:**
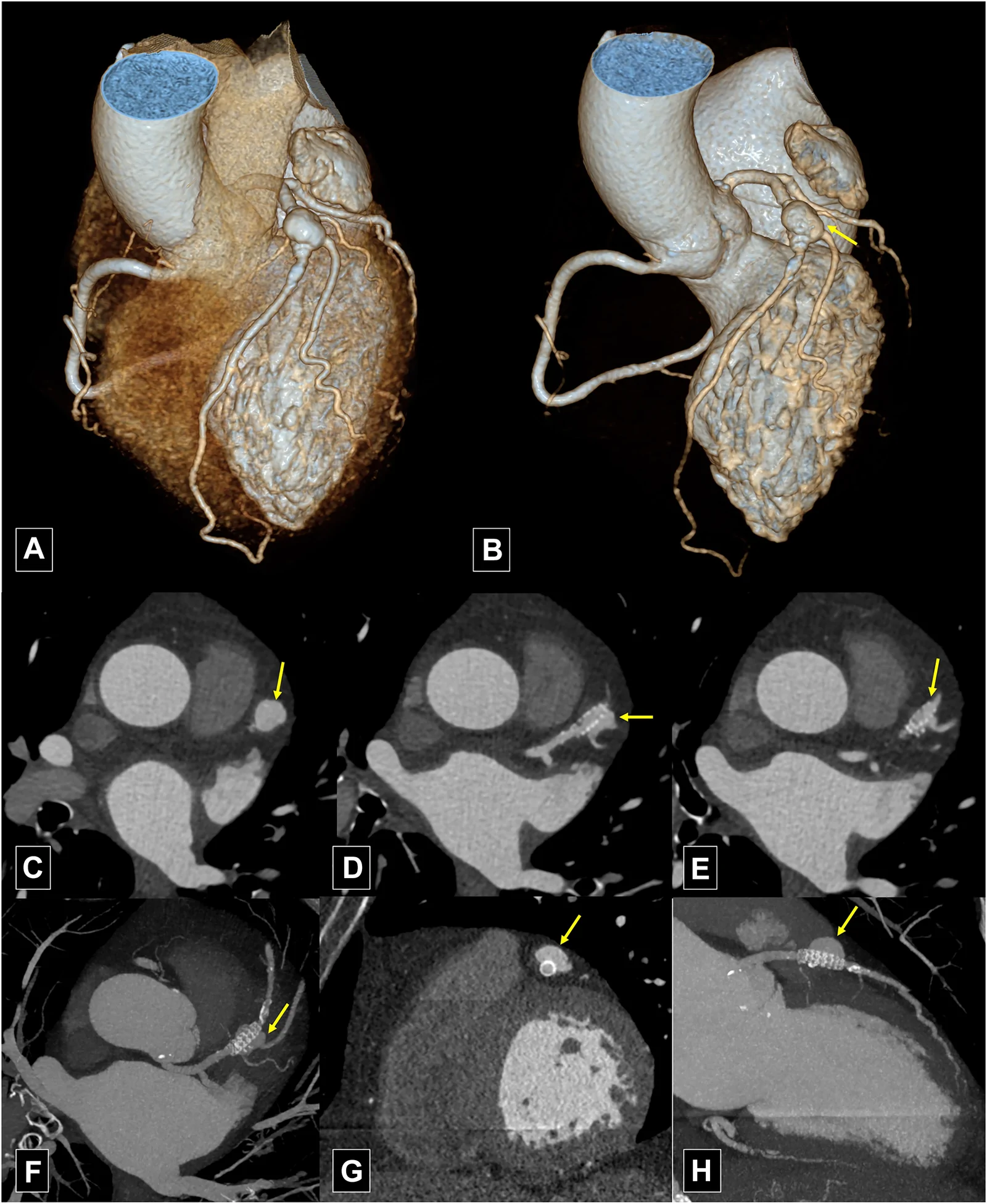
Preoperative Computed Tomography (3D Reconstruction) Preoperative coronary computed tomography angiography images (volume-rendered projections A and B, axial sections C to E, and multiplanar reformats F to H), depicting the left anterior descending coronary artery aneurysm (yellow arrows).

## Management

Given her new symptoms, evidence of ischemia, and aneurysm size, aneurysm repair was decided and executed. The pulmonary artery was transected (Figure 4) to expose the aneurysm and the left main trunk. The aneurysm was incised longitudinally, and the stent removed exposing septal perforators at the distal and proximal end of the aneurysm and the 3 diagonal branches (2 small and 1 larger) coming off the aneurysm. LAD continuity was restored with interposition of a saphenous vein graft between the left main and LAD. The diagonal side branches were reimplanted to the side of the vein graft. Calcification of the aneurysm wall increased complexity, but we were able to achieve adequate hemostasis.

**Figure 4:**
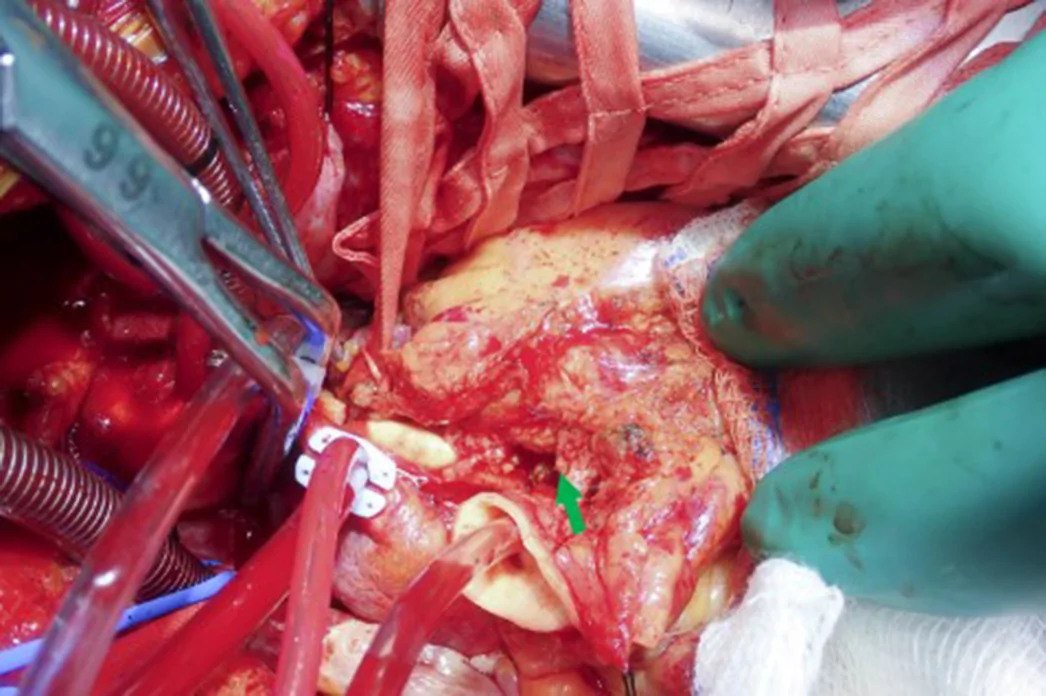
Pulmonary Artery Transection to Further Expose Left Main Coronary Trunk and Aneurysm (Green Arrow)

## Outcome and Follow-Up

The patient was extubated a few hours after the procedure, transferred to stepdown on postoperative day 1, and discharged on postoperative 5. Before discharge, computed tomography angiography verified a good repair, visualizing an LAD wrapping around the apex and intact septal and 2 diagonal branches (Figure 5). One month after the surgery, she remained clinically stable with no new cardiac complaints and adherent to medication, dietary, and rehabilitation programs.

**Figure 5:**
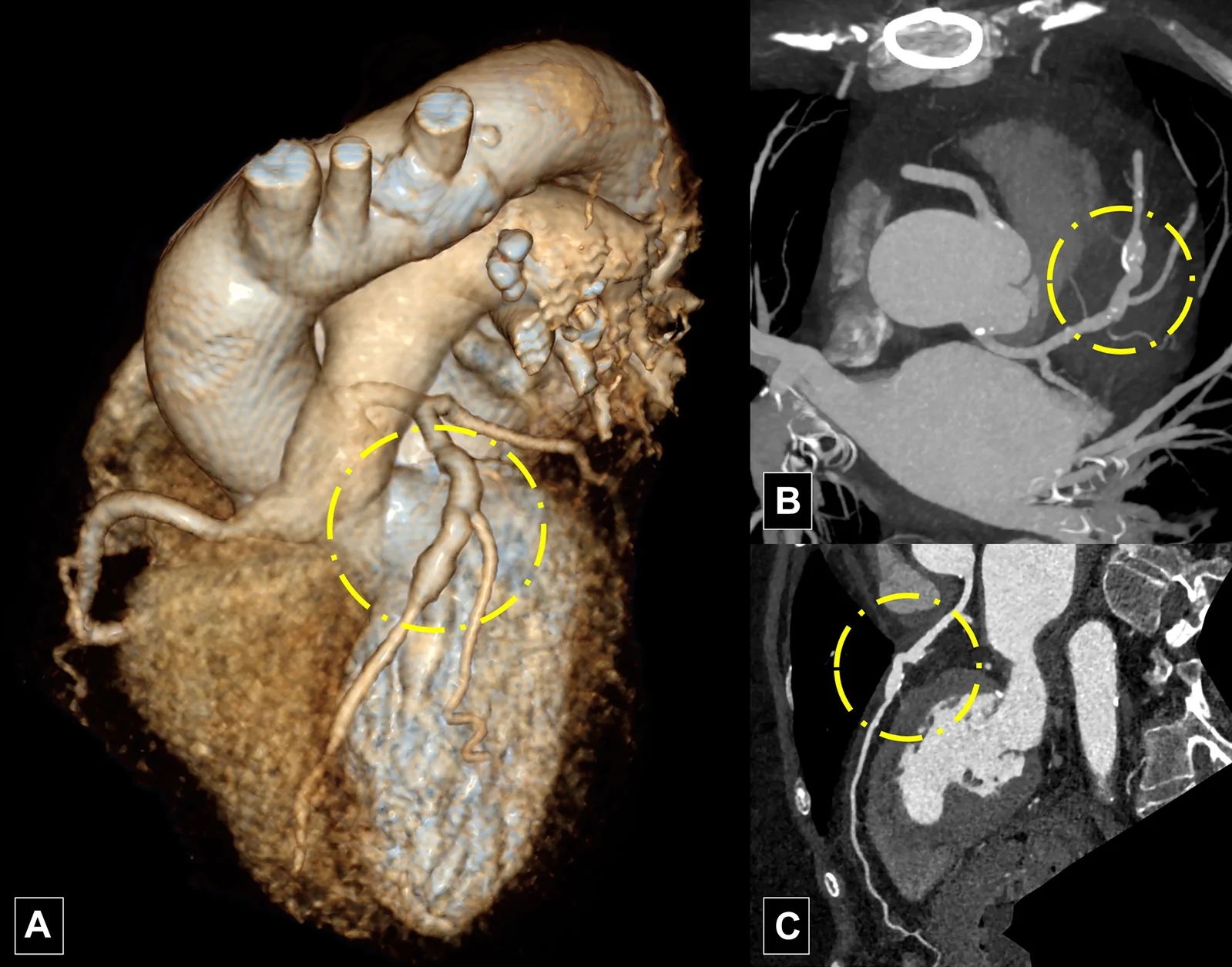
Postoperative Computed Tomography Angiography Images Volume-rendered projection (A) and multiplanar reformats (B and C) depicting the repair. The yellow circle shows the patent vein graft.

## Discussion

Based on the literature, a diameter >20 mm, 40 mm, 50 mm, or quadruple the reference vessel diameter is considered a giant coronary artery aneurysm (CAA).^1^ In this case, the CAA measured 13 mm. Although CAAs have been reported from 3 days to up to 4 years after DES,^2^ this aneurysm presents many years after implantation of a DES and has grown in size over a period of 4 years. In 2004, our patient received a first-generation DES with stainless steel structure coated with sirolimus and polymer 1, which 6 years later occluded resulting in myocardial infarction, requiring PCI plus atherectomy. The history of previous DES and percutaneous transluminal coronary angioplasty are likely to have triggered the late aneurysmal development in our patient.^3^ Tracheal and breast radiotherapy may have also contributed to weakening of the coronary wall leading to aneurysm.^4^

CAAs are uncommon, with a reported incidence of 0.14% to 4.9% among patients undergoing coronary angiography and 0.3% to 5.3% following percutaneous transluminal coronary angioplasty.^5^ CAAs are often associated with coronary atherosclerosis and present in 0.3% to 12% of patients undergoing angiography.^5^ They are usually asymptomatic but can be a cause of ischemia and may be complicated by thrombosis (up to 4.8%) and rupture (0.2%).^6^ Our patient’s acute chest pain, stress test, and positron emission tomography findings suggested ischemia in the LAD territory.

When indicated by size, ischemia, infection, or other related issues, CAAs can be managed percutaneously with stents or coil embolization or surgically, the most common surgical procedures being ligation, followed by coronary artery bypass grafting.^6^^,^^7^ Resection and end-to-end anastomosis also have been described.^8^ In our case, proximal ligation and bypass distally to LAD was discussed, but that option would likely have sacrificed septal and small diagonal arteries. An alternative new approach is to do an interposition graft and create a bypass within the aneurysm at the ostium of the branch.^9^^,^^10^ The principle relies on opening the entire aneurysm and reconstructing the LAD within the aneurysm with an interposition saphenous vein graft.

The technique of choice was not only influenced by the aneurysm size, but also the location and more importantly, the number and size of the branches coming out of the aneurysmal segment. We aimed to achieve as much of a complete revascularization as possible to minimize the risk of arrythmias and circulatory support. In our patient, the diagonal branches were too small to perform epicardial bypasses. This technique would have likely sacrificed these branches. Our experience with treating aneurysmal coronary arteries with fistulas taught us 2 lessons: 1) it is easier to identify branches that may not be apparent on the epicardial surface and 2) it is technically easier to graft small branches at their intracoronary ostia.

Excluding the aneurysm and attempting to do epicardial bypasses would have been limited to the small size of the diagonal branches and septal perforator, likely leading to incomplete revascularization. This may have been evidenced immediately in the operating room with difficulty weaning off pump or postoperatively with arrythmias. Although long-term patency is lower in saphenous vein grafts than their arterial counterparts, shorter saphenous vein grafts have better patency.

Although reimplanting the diagonal branches to the side of the vein as a button was no problem from a size standpoint, the wall calcifications in the aneurysm added some difficulty. A proximal and a distal septal perforator were left outside the anastomoses and preserved. We opted for a venous graft because of the size and pliability. It has the closest size to the native LAD, whereas arterial grafts are smaller at the time of grafting and need time to mature. It allowed accommodation of larger diameter distal vessels and covered multiple diagonal branches. Veins are usually more pliable and can give us more freedom to perform this type of procedure.

In addition, we use internal thoracic arteries for important reachable single vessels instead of complicated cases requiring multiple side-to-side anastomoses. Short interposition veins with multiple anastomoses and good runoff should have patency. They may even show arterialization like internal thoracic arteries with vein extensions. The main disadvantages of veins that sometimes make the decision difficult are seen when they kink, lay, and are large.

## Conclusions

Although more complex, in situ coronary aneurysm repair provides a physiological repair, with abolishment of the aneurysm and complete revascularization.

**Visual Summary:**
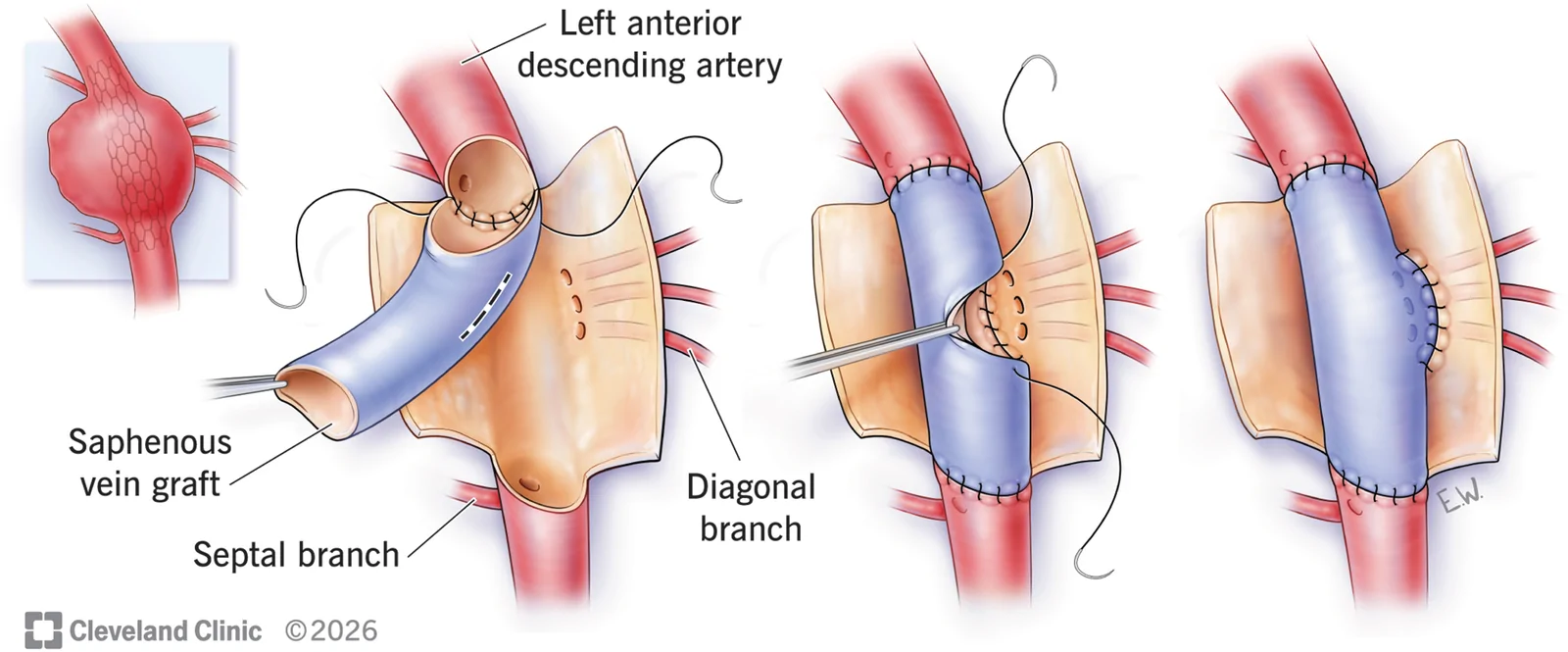
Repair of Coronary Aneurysm With Interposition of Saphenous Vein Graft Re-Establishes Flow to the Main Vessel and Tributaries Adequate exposure and meticulous techniques are essential for success.

## Funding Support and Author Disclosures

Dr Roselli has received speaking honoraria from Artivion (formerly CryoLife/Jotec), Edwards Lifesciences and W.L. Gore and Associates. All other authors have reported that they have no relationships relevant to the contents of this paper to disclose.

**Take-Home Messages**

• Repair of coronary aneurysm with interposition of saphenous vein graft re-establishes flow to the main vessel and tributaries.
• Adequate exposure and meticulous techniques are essential for success.
